# Trends and burden of tobacco use in Nepal: Insights from the Global Burden of Disease study 1990–2021

**DOI:** 10.18332/tpc217390

**Published:** 2026-03-19

**Authors:** Kiran Paudel, Kamal Gautam, Sandhya Niroula, Sandesh Bhusal, Jeffrey A. Wickersham, Safaet Hossain Sujan, Bhakta KC, Tara B. Adhikari, Roman Shrestha

**Affiliations:** 1 Nepal Health Frontiers, Kathmandu, Nepal; 2 Department of Allied Health Sciences, University of Connecticut, Storrs, United States; 3 Department of Internal Medicine, Section of Infectious Diseases, Yale School of Medicine, New Haven, United States; 4 Institute of Medicine, Tribhuvan University, Kathmandu, Nepal; 5 Ministry of Health and Population, Government of Nepal, Kathmandu, Nepal; 6 Research Unit for Global Health, Aarhus University, Aarhus, Denmark

**Keywords:** DALYs, Global Burden of Disease (GBD), burden, smoking tobacco, Nepal

## Abstract

**INTRODUCTION:**

Tobacco consumption is a significant public health problem in Nepal, accounting for 39200 deaths each year, accounting for 23.7% of all deaths. While Nepal has taken several policy measures to strengthen tobacco control, the persistent burden of tobacco and the associated health risks highlight the need for continuous monitoring and evaluation. Therefore, this study assessed the trend in age-specific and sex-specific mortality and disability attributable to different forms of tobacco use (smoking, chewing tobacco) in Nepal from 1990 to 2021.

**METHODS:**

This observational study used publicly available data from Nepal's Global Burden of Disease (GBD) 2021 estimations. The age-standardized and age-specific summary exposure value (SEV), mortality, and disability-adjusted life years (DALYs) were extracted to measure the burden and trend of tobacco use. The data are presented as percentages or rates per 100000 population.

**RESULTS:**

From 1991 to 2021, the age-standardized SEV of tobacco consumption for both sexes at all ages decreased from 44.1% to 28.2%. The age-standardized deaths attributable to tobacco use, including all forms of tobacco products, decreased by (46.7%) from 262.9 (95% UI: 193.5–344.3) per 100000 in 1990 to 140.2 (95% UI: 101.1–181.9) per 100000 in 2021.

**CONCLUSIONS:**

Despite declines in SEV, and DALYs from 1990 to 2021, tobacco use remains a major public health concern. Strengthening smoking cessation programs, enforcing stricter tobacco control policies, raising taxes on tobacco products, and expanding public awareness campaigns are essential to reducing its burden.

## INTRODUCTION

Tobacco use is a major global health threat, killing over 8 million people each year, including more than 7 million from direct use of smoked tobacco and smokeless tobacco or chewing tobacco and 1.3 million from secondhand smoke exposure^1,2^. It kills more than any other preventable cause of death in the world^3^. Tobacco use is attributed to be a leading risk factor for the rising burden of non-communicable diseases (NCDs) such as cardiovascular diseases (e.g. heart attack, stroke), cancers, chronic respiratory diseases (e.g. chronic obstructive pulmonary disease, asthma) and diabetes^4,5^. One in six deaths by NCDs is related to tobacco use^3,6^. Around 80% of the world’s 1.3 billion tobacco users reside in low- and middle-income countries (LMICs), where 80% of tobacco-related deaths occur^1,7^. The economic cost of tobacco use is estimated to be around $1.85 trillion globally, making it a total of about 1.8% of the world’s GDP^8^.

In Nepal, tobacco use is responsible for an estimated 39200 deaths each year, accounting for 23.7% of all deaths^9^. Of these, 24100 deaths (85.7%) are attributable to smoking, while 4500 deaths (16.2%) result from exposure to secondhand smoke^10^. According to recent findings from the STEPwise Approach to Surveillance (STEPS) survey 2019 in Nepal, approximately 29% of adults (48% male and 12% female) within the age group 15–69 years used any form of tobacco. In Nepal, current (either daily or occasionally at the time of survey) smokeless tobacco use (overall: 18.3%, males: 33.3%, females: 4.9%) is more prevalent than tobacco smoking (overall: 17.1%, males: 28.0%, females: 7.5%), with higher usage observed among males in both categories^11^. Various forms of smokeless tobacco (SLT) are consumed, like gutkha (contains areca nut, tobacco, catechu, and sweet flavor), zarda paan or betel quid (rolled betel leaf with lime, betel nut, and tobacco), and khaini (flavored tobacco mixed with lime)^12,13^.

The World Health Organization (WHO) Global Action Plan for the Prevention and Control of Noncommunicable Diseases (NCD-GAP) set a target of a 30% relative reduction in tobacco use by 2025, using 2010 as the baseline^14^. However, projections suggest that global tobacco prevalence will reach 19.8% in 2025 and 18.1% by 2030. These figures fall short of the targets needed to achieve the goal^15^. Despite overall progress, the decline in tobacco use has not been as rapid as anticipated, and significant regional disparities persist^14,15^. In particular, tobacco use remains disproportionately high in LMICs, including Nepal^15^. While many countries have successfully implemented tobacco control policies such as MPOWER (Monitoring, Protecting people from tobacco smoke, Offering help to quit tobacco use, Warning about dangers of tobacco, Enforcing bans on advertising, promotion and sponsorship, and Raising taxes on tobacco), progress has been uneven^15^.

Nepal has taken several policy measures to strengthen tobacco control^16^. The country ratified the WHO Framework Convention on Tobacco Control (WHO FCTC) in 2006^15^. It passed the Tobacco Control and Regulatory Bill in 2011^16^, aiming to reduce tobacco consumption through stricter regulations and public health interventions. Despite these efforts, the ongoing burden of tobacco use and its health risks highlight the need for continuous monitoring and evaluation^17^. A comprehensive assessment of the long-term burden of tobacco use is essential to understand trends, measure progress, and inform evidence-based policies. Updated knowledge of the current pattern of tobacco use will be beneficial for policymakers to curb the tobacco epidemic. This study systematically reviewed the data extracted from the Global Burden of Disease (GBD) Study 2021. It assessed the trends in prevalence, mortality, and disability attributable to different forms of tobacco in Nepal from 1990 to 2021. The study findings will support the development of effective strategies for reducing tobacco-related harm and improving public health outcomes in Nepal.

## METHODS

### Study design

This observational study on the burden of tobacco use was based on secondary data obtained from the GBD study 2021 estimations for Nepal^18^.

### Data sources and study settings

The Institute for Health Metrics and Evaluation (IHME) coordinated the GBD study 2021^19^. This comprehensive epidemiological study reports the morbidity and mortality pattern in 195 countries, ranging from significant injuries, diseases, and risk factors to health. GBD study 2021 used surveillance data, national surveys, epidemiological studies, several published and unpublished literature, and hospital and clinic data to estimate the burden of 84 risk factors for 195 countries by age and sex^18,20^. The GBD visualization tool and study protocol are available online. Studies have already reported the applied methods of GBD 2021 earlier^4,17^. We downloaded the SEV, mortality rate, DALYs, YLLs (years of life lost), and YLDs (years lived with disability) data of tobacco, smoking, secondhand smoking, and smokeless tobacco for Nepal by age and sex from 1990 to 2021 in an Excel format.

The study is exempt from ethical review and approval as it used publicly available de-identified data from the IHME’s GBD database.

### Statistical analysis

Data were obtained from the IHME and imported into Microsoft Excel, then quantitatively analyzed and presented in graphical and tabular forms. A percentage change was calculated to demonstrate the difference between 1990 and 2021. The 95% uncertainty interval (UI) is used to show the strength of the estimates. The higher the gaps, the lower the strength of the estimates^21^. The UI is a range of values that is likely to include correct estimates of disease burden for a given cause^21^.

DALYs and mortality (death) are the most common measures that GBD uses to estimate the Burden of Disease (BoD). DALYs are calculated by combining YLLs and YLDs. YLLs are determined by multiplying the number of deaths at each age by the standard life expectancy. It refers to the years of life lost due to premature mortality. YLDs measure the time spent living with a health condition, adjusted by the severity of its impact on daily life^20,21^.

The summary exposure value (SEV) measures the population’s exposure to a risk factor, considering the extent of exposure by risk level and the severity of that risk’s contribution to disease burden^21^.

## RESULTS

**Figure 1:**
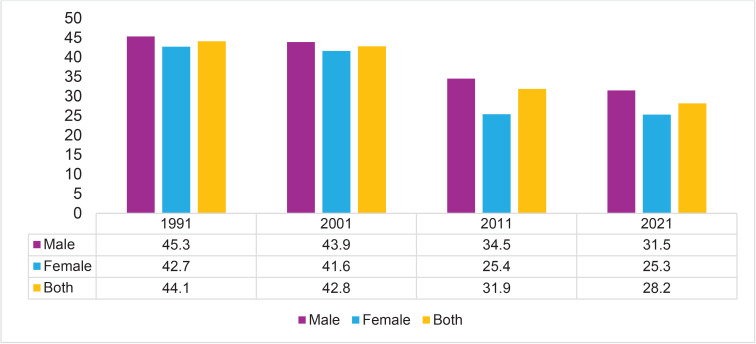
Trend in the summary exposure value of tobacco consumption, by gender, in Nepal from 1991 to 2021

The trend of the summary exposure value of tobacco consumption is decreasing from 1991 to 2021 in both sexes. In 1991, the age-standardized SEV of tobacco smoking at all ages was 44.1%. The SEV was more prevalent among males (45.3%) than females (42.7%). In 2021, the SEV of tobacco use decreased by 28.2% from 44.1% in 1991 for both sexes at all ages, as shown in Figure 1.

**Figure 2:**
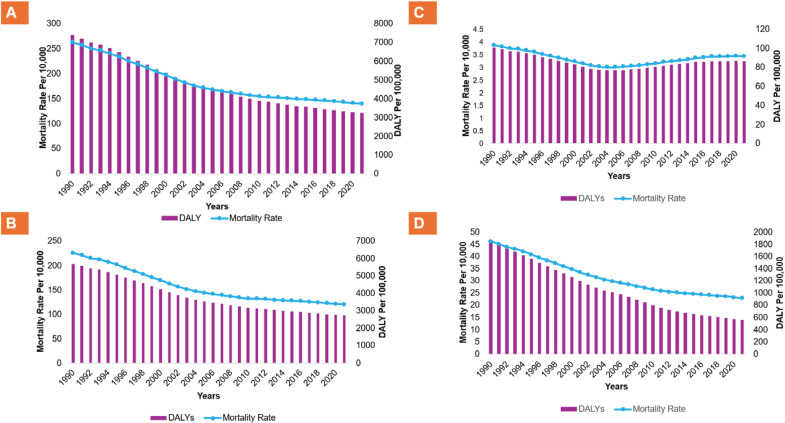
Trend in age-standardized mortality and DALYs rate in Nepal from 1990 to 2021, from: A) Overall tobacco use, B) Smoking tobacco, C) Chewing tobacco, and D) Secondhand smoke

In Nepal, both the age-standardized mortality rate and the DALYs attributable to tobacco products and secondhand smoking are in a decreasing trend from 1990 to 2010, and their overall burden has remained relatively stagnant from 2010 to 2021 as shown in Figure 2. The age-standardized deaths attributable to it, including all forms of tobacco products, decreased by (46.7%) in the general population from 262.9 (95% UI: 193.5–344.3) per 100000 in 1990 to 140.2 (95% UI: 101.1–181.9) per 100000 in 2021. Similarly, DALYs decreased by 56.1% from 7404.3 (95% UI: 5311.2–9853.6) per 100000 in 1990 to 3250.5 (95% UI: 2352.6–4195.2) per 100000 in 2021 as shown in Table 1. Similar trends were observed in both males and females and for all forms of tobacco products (i.e. smoking and chewing tobacco), and secondhand smoke exposure, as shown in Table 1.

**Table 1:** Age-standardized deaths and DALYs for different diseases attributable to tobacco use and their percentage change in Nepal from 1990 to 2021

*Causes*	*Age-standardized death rate per 100000 (95% UI)*			*Age-standardized DALYs rate per 100000 (95% UI)*		
*All*	*1990*	*2021*	*% Change*	*1990*	*2021*	*% Change*
**Tobacco**						
Male	312.3 (223.4–408.4)	186.9 (138.4–238.1)	-40.1	8610.2 (6340.1–11339.9)	4362.1 (3241.1–5619.2)	-49.3
Female	212.4 (149.2–287.7)	99.4 (65.8–136.3)	-53.2	6146.9 (4132.2–8384.1)	2275.9 (1530.2–3062.4)	-62.9
Both	262.9 (193.5–344.3)	140.2 (101.1–181.9)	-46.7	7404.3 (5311.2–9853.6)	3250.5 (2352.6–4195.2)	-56.1
**Smoking tobacco**						
Male	279.6 (210.2–361.5)	166.1 (125.9–211.0)	-40.6	7048.2 (5329.6–9127.5)	3808.5 (2871.8–4854.3)	-45.9
Female	171.4 (120.9–232.7)	80.7 (54.6–112.5)	-52.9	4325.8 (3129.8–5736.5)	1901.3 (1252.2–2430.9)	-56.1
Both	226.1 (168.3–295.6)	120.5 (89.1–155.2)	-46.7	5715.0 (4324.9–7493.1)	2739.6 (2038.1–3493.7)	-52.1
**Chewing tobacco**						
Male	4.5 (2.9–6.3)	4.3 (2.9–6.2)	-4.4	124.5 (80.2–178.0)	118.8 (76.8–170.2)	-4.6
Female	3.2 (2.1–4.4)	2.7 (1.8–3.7)	-15.6	75.2 (51.6–103.6)	58.6 (38.5–82.9)	-22.1
Both	3.9 (2.8–5.1)	3.4 (2.4–4.5)	-12.8	100.6 (70.6–134.3)	86.5 (60.0–116.5)	-14.0
**Secondhand smoke**						
Male	42.8 (19.1–70.7)	25.5 (12.5–39.6)	-40.4	1749.1 (667.8–2885.8)	624.7 (307.3–966.2)	-64.3
Female	49.9 (22.9–79.9)	20.9 (9.5–32.8)	-58.1	1999.7 (807.3–3211.5)	506.7 (224.6–798.7)	-74.7
Both	46.3 (21.0–73.8)	23.0 (10.9–35.7)	-50.3	1871.9 (729.7–3023.5)	561.4 (262.1–884.7)	-70.0

**Figure 3:**
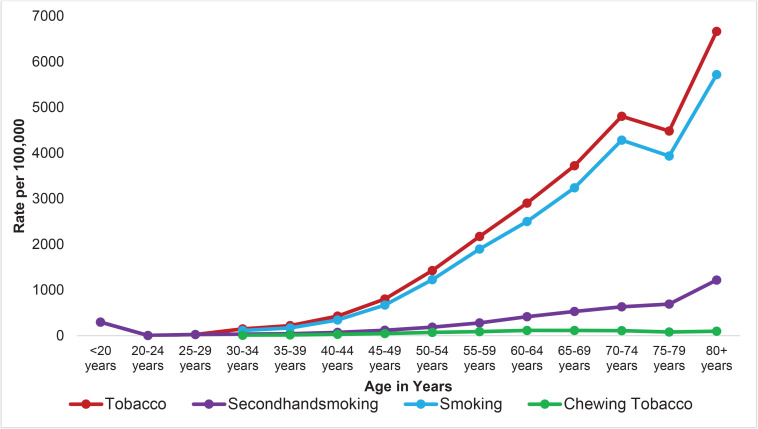
Mortality rate from overall tobacco, smoking tobacco, chewing tobacco, and secondhand smoke exposure, by age, in Nepal, 2021

**Figure 4:**
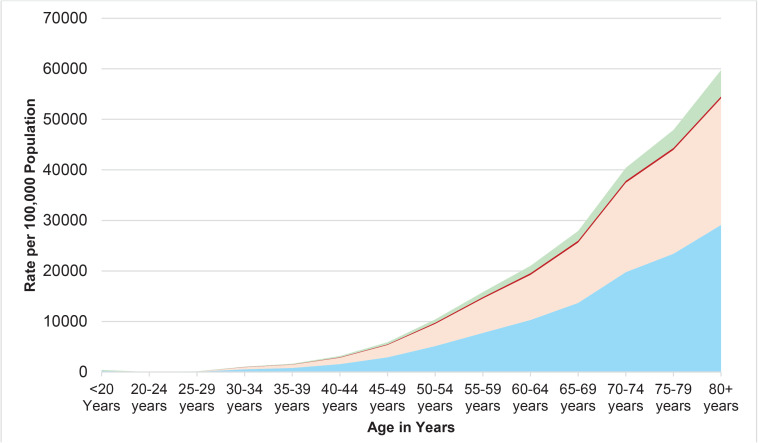
DALYs rate from tobacco, smoking tobacco, chewing tobacco, and secondhand smoke exposure, by age, in Nepal, 2021

Figures 3 and 4 illustrate age-related increases in the rates of deaths and DALYs attributable to tobacco use in 2021. In contrast, secondhand smoke exposure and chewing tobacco demonstrate relatively stable patterns across age groups, without sharp upward trajectory, as observed for active smoking.

**Figure 5:**
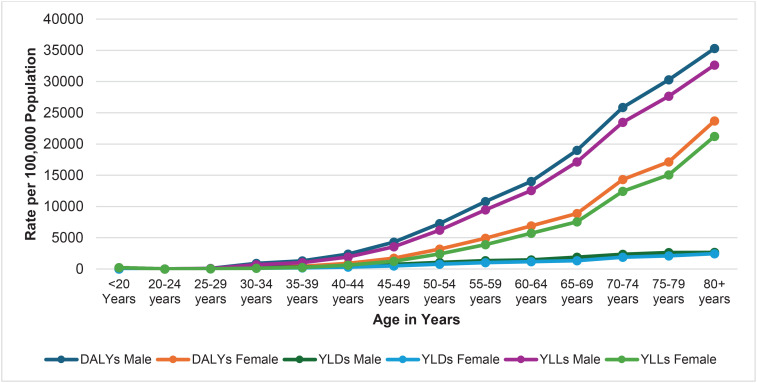
Tobacco-related DALYs, YLLs, and YLDs, by age and sex, in Nepal, 2021

In 2021, the DALYs and YLLs for males demonstrate a sharp increase with increasing age, a trend that was similarly evident in females for tobacco-related outcomes. However, the YLDs for both males and females do not demonstrate a similarly steep upward trend as seen with DALYs and YLLs, as shown in Figure 5.

In absolute numbers, the number of deaths attributable to tobacco use among people of all ages increased in the general population from 24727.5 (95% UI: 17590.6–33193.0) in 1990 to 28110.2 (95% UI: 20351.7–36598.9) in 2021. The increasing trend is similar for smoking and chewing tobacco, as shown in Supplementary file Figure 1. The age standardized rates decline while absolute count can rise due to population growth or ageing.

## DISCUSSION

The GBD study results indicate that between 1990 and 2021, the burden of tobacco like SEV, and DALYs decreased. One of the major reasons for the decrease in the burden of tobacco use in Nepal could be attributed to the implementation of various policy measures over the last two decades, including the WHO FCTC in 2006^14,22^, the Tobacco Control and Regulatory Bill 2011^16,23^, and other anti-tobacco campaigns and policies^16^, as shown in Supplementary file Figure 2.

Our findings indicated that the burden of tobacco use was more prevalent among males compared to females. This is consistent with the findings from the Noncommunicable Disease Risk Factors: WHO STEPS survey, Nepal 2019, which reported that smoking among males was 48.3% and 11.3% in females^11^. One of the reasons for this could be the social acceptance for tobacco use for men but not so much for women in Nepal. This could also lead to the underreporting of tobacco use in females. In addition, the patriarchal norms might have encouraged men to use tobacco as a symbol of masculinity or social status. Another possible reason may be that males are more likely to frequently engage in social gatherings where tobacco use is common. Existing evidence has shown a similar pattern in other LMIC settings, such as the Philippines, Indonesia, the Maldives, Bangladesh, Vietnam, and Malaysia^24-28^. Tobacco cessation programs and other interventions should be tailored according to the gender in order to address these differences.

The study revealed that the deaths and DALYs due to tobacco use increased with increasing age. This might be due to the harmful effects of smoking accumulation over time, which might have damaged the lungs, heart, and other body parts. Deposition of tobacco-related chemicals in the body might have caused lung cancer, chronic respiratory diseases, and other NCDs^29^, which would increase DALYs and death rates with increasing age. This finding aligns with studies conducted in Nepal and other countries^16,17,26^. Older adults have different beliefs that continuing tobacco use does not harm and stopping tobacco use does not improve health status^30^.

Nepal has made significant progress in tobacco control, particularly in policy measures related to warning labels, public smoking bans, and advertising restrictions^16^. A sharp decline in tobacco-related mortality and DALYs was observed until 2010, with no considerable progress after that. However, the absolute number of tobacco-related deaths continues to rise due to population growth and continued tobacco use. While Nepal has excelled in implementing key aspects of the WHO MPOWER framework – such as health warnings, smoke-free environments, and advertising bans – there remain significant gaps in offering help to quit tobacco use^31^. In 2022, Nepal used about 1.2 thousand hectares of land for tobacco farming, which is 53.7% less than in 2010^9^. This shows that tobacco control has been effective. However, tobacco control progress in Nepal has plateaued in recent years, despite existing laws and policies. Loopholes in regulations allow continued tobacco use, underscoring the need for stricter enforcement and expanded cessation programs.

Nepal has maintained 100% pictorial health warnings on cigarette packs^32^. However, it lags in systematic monitoring^31^. Tobacco taxation in Nepal remains significantly lower (31.4%) than the WHO-recommended rate of 75%, indicating that current tax levels are insufficient and should be increased accordingly^31^. Despite the rise in tobacco taxes to 31.4%, the affordability of cigarettes has not decreased, suggesting that the existing tax structure may still be inadequate to effectively curb tobacco consumption. This is because income growth surpasses the small tax increases, making tobacco more affordable. An increase in taxes could potentially lead to an increase in illicit trade, particularly due to the open border with India. Concerns about illicit tobacco trade may partly explain why taxes have not yet been raised to the WHO-recommended levels. Nevertheless, the observed decline in the tobacco burden likely reflects the positive impact of recently enacted policies^33^.

The tobacco cessation program in Nepal faces significant challenges due to limited resources and infrastructure^16^. For example, the limited availability of a cessation program (e.g. nicotine replacement therapy), currently concentrated in two government hospitals within the Kathmandu Valley, suggests a gap in accessible treatment for tobacco dependence, particularly in rural areas^10^. Doctors can help smokers quit, but Nepal has very few doctors compared to its population^34^. Due to limited time and resources, doctors often focus on treating urgent and severe conditions such as heart attacks, strokes, and lung cancer, even though smoking is a major cause of these diseases. Research shows that even brief advice from doctors can help patients quit smoking, and follow-up support makes it even more effective^35^. This approach can also work with other healthcare workers^36^, not just doctors. In Nepal, preferably psychiatrists can prescribe smoking cessation medicines like varenicline and bupropion^37^. We can involve other healthcare workers in tobacco cessation efforts by providing them with appropriate training and revising relevant policies and guidelines. This approach would help to make tobacco cessation programs more accessible and widely available. The effectiveness of the program could be enhanced if local governments took the responsibility. For broader awareness and prevention, Female Community Health Volunteers (FCHVs), who are the cornerstone of community health system in Nepal, can play a vital role and should be actively engaged. FCHVs have successfully improved many health indicators like maternal health, hypertension, and diabetes in communities^38^. Similarly, FCHVs could be trained to support smoking cessation efforts as part of tobacco cessation campaigns. It is time for Nepal to focus on the equation (Impact = Effectiveness × Reach)^39^. This shows how interventions achieve population impact, but real-world results may differ^39^. In practice, individuals such as smokers may be less motivated or require additional promotion, resulting in outcomes that differ from those observed in research trials^39^. To reduce the ongoing burden of tobacco use, which has remained constant from 2010 to 2021, Nepal needs to implement strong and urgent measures like the above.

### Limitations

This is one of the few studies to estimate the burden of tobacco in Nepal using the GDB 2021 study, which draws on exhaustive information sources and comprehensive investigations. Despite its importance, this study has some limitations which need to be acknowledged. First, the study relies on secondary data, some of which are derived from publicly available sources that may not fully represent the entire nation. Second, the burden of diseases was estimated based on epidemiological measures without considering other economic and social burdens. Due to limited data in the GBD study, we were unable to analyze the results separately for different provinces or socioeconomic groups. Third, Nepal lacks a national system to track diseases; thus, the GBD study has to rely on mathematical predictions rather than actual local data. Finally, these models group all smokeless tobacco products together, which may not capture the higher toxicity of local products. This means the current estimates could be either overestimating or underestimating the true health burden.

## CONCLUSIONS

Despite the decreasing trend in SEV, and DALYs from 1990 to 2021, tobacco use remains a significant public health concern in Nepal. These findings underscore the need for a comprehensive tobacco control strategy that fully implements and enforces all components of the WHO MPOWER framework, with a specific focus on the WHO’s best buys. Priority actions must include increasing excise taxes on all tobacco products, ensuring 100% smoke-free indoor workplaces, and implementing effective public education campaigns. Current efforts remain insufficient to achieve substantial reductions in tobacco use and its associated health burden.

## Supplementary Material



## Data Availability

This study is based on publicly available data from the Global Burden of Disease 2021. The data can be downloaded from https://vizhub.healthdata.org/gbd-results/ . If the data are not available from this link, the corresponding author can provide access upon reasonable request.
