# Supplementary material for: Trends and burden of tobacco use in Nepal: Insights from the Global Burden of Disease study 1990–2021
Source: Tob Prev Cessat. 2026 Mar 19;12:10.18332/tpc217390. doi: 10.18332/tpc217390 (PMC13001751; doi:10.18332/tpc217390)
Supplement: Supplementary file 1 [file TPC-12-16-s1.pdf]

Supplementary File

Figures

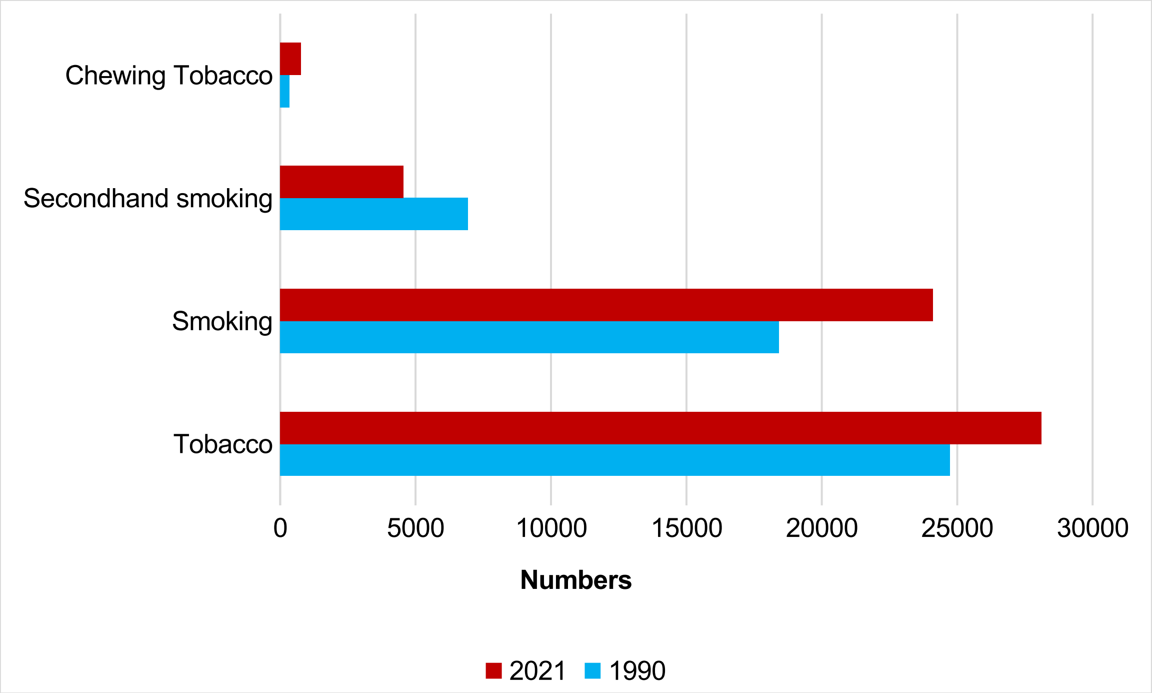

Supplementary Figure 1: Attributable absolute number of deaths from tobacco use in 1990 and 2021, including smoking, secondhand smoke exposure, and chewing tobacco, across all age groups

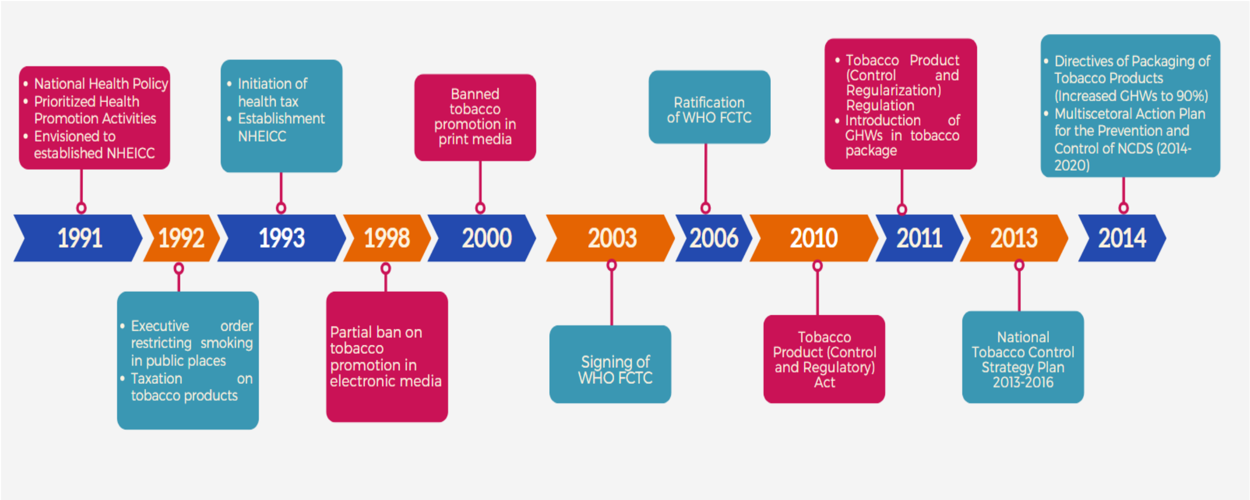

Supplementary Figure 2: Major milestones in tobacco control and regulation from the 1990s in Nepal

© 2026 Paudel K. et al.
